# Space and players' number constrains the external and internal load demands in youth futsal

**DOI:** 10.3389/fspor.2024.1376024

**Published:** 2024-05-28

**Authors:** Sérgio Adriano Gomes, Bruno Travassos, João Nuno Ribeiro, Henrique de Oliveira Castro, Leandro Lume Gomes, Carlos Ernesto Santos Ferreira

**Affiliations:** ^1^ Physical Education Department, Universidade Católica de Brasília, Brasília, Brazil; ^2^ Secretaria de Estado de Educação do Distrito Federal, Brasília, Brazil; ^3^ Sport Science Department, Universidade da Beira Interior, Covilhã, Portugal; ^4^ CIDESD, Centro de Investigação em Desporto, Saúde e Desenvolvimento Humano, Covilhã, Portugal; ^5^ Portugal Football School, Federação Portuguesa de Futebol, Oeiras, Portugal; ^6^ Polytechnic Institute of Guarda, School of Education, Communication and Sports, Guarda, Portugal; ^7^ SPRINT Sport Physical Activity and Health Research & Innovation Center, Portugal; ^8^ Physical Education Department, Universidade Federal de Mato Grosso, Cuiabá, Brazil; ^9^ Physical Education Faculty, Universidade de Brasília, Brasília, Brazil

**Keywords:** team sports, small-sided games, ecological dynamics approach, training & development, load management and load response

## Abstract

**Introduction:**

The aim of this study was to analyze the effects of space and number of players manipulation on the external and internal load demands of youth futsal athletes.

**Methods:**

Forty-two male U17 players (age = 15.62 ± 0.58 years) from three futsal teams participated in the study. In this cross-sectional study that lasted 8-week, the player's sample practiced six futsal tasks (T1–T6) and a futsal game played under the official rules (T7). From T1–T6, two task constraints were manipulated: (i) the number of players and, (ii) the space of play. The WIMU PRO™ Ultra-Wideband (UWB) tracking system was used to measure the external and internal load during the futsal tasks. External load was quantified using kinematic and mechanical variables extracted from positional data and, the internal load was quantified using Heart rate (HR) and rating of perceived exertion (RPE). Repeated measures ANOVA was used for comparison purposes.

**Results:**

In general, the results showed high external (total distance, distance 18.1–21, above 21 Km/h, and high intensity acceleration and deceleration, *p* < 0.001) and internal load (heart rate average and rating of perceived exertion, *p* < 0.001) in the tasks with low number of players and high area. In relation to the match, the tasks with small relative area per player (GK + 2 vs. 2 + GK and GK + 3 vs. 3 + GK in 20 × 20 m) promoted low external load.

**Conclusion:**

It was concluded that increasing the relative area by reducing the number of players involved in the tasks in the form of small-sided games (GK + 2 vs. 2 + GK and GK + 3 vs. 3 + GK), in relation to the futsal game (GK + 4 vs. 4 + GK), can be considered a pedagogical strategy to increase the external and internal load demands of young futsal players.

## Introduction

In futsal, external and internal loads are used to monitor and control training and match demands (1–4). Studies have investigated the external load demands of futsal using tracking technology (3, 4, 5–8). On average, male player's covers a distance of 4,000 meters (m) during official futsal matches, corresponding to 120 m/min^−1^. The distances covered include walking (397 meters), jogging (1.762 m), medium-intensity (1.232 m), high-intensity (571 m), and maximum speed running (349 meters) (9, 10). Meanwhile, Serrano et al. (7) found no significant differences in overall physical performance between the first and second halves, although differences in high-intensity actions were noted between pivot and wing players. In terms of internal load, it is common to quantify the rating of perceived exertion (RPE) in response to exercise engagement (1). The session-RPE method has proven to be effective in quantifying internal training loads, not only in general exercise contexts (11–13), but also specifically in futsal (1, 14–16). Clemente et al. (2) recently conducted a study on elite U20 futsal players and found a moderate negative correlation between external/internal training load, muscular soreness and fatigue. Meanwhile, Chen et al. (1) showed that responses to training sessions in elite futsal players, as derived from session-RPE and wellness scores, are task-dependent.

Understanding and quantifying external and internal load demands in futsal provides important information for coaches and may be useful in designing appropriate training programs (17). For instance, Pizarro et al. (18) analyzed the effects of number of players and floater position on offensive performance during small-sided games (SSGs) in U19 male futsal players. The study revealed notable distinctions between 2 vs. 2 and 3 vs. 3, highlighting the impact of player numbers on team tactical behavior. Furthermore, Rigon et al. (19) investigated the effect of numerical player configuration and court size restrictions on the difficulty of futsal SSGs, concluding that larger spaces led to reduced player participation. To replicate the demands of the match and promote the necessary adaptations in futsal, coaches typically manipulate the areas of play (e.g., 20 × 20 m, 30 × 20 m, and 40 × 20 m) and the number of players involved (e.g., 2 vs. 2, 3 vs. 3, and 4 vs. 4) (20). These modifications require players to adapt to new game scenarios with different situational contexts and game demands (21, 22). However, these manipulations not only promote individual tactical adaptations in players actions but also promote variations in physiological and physical stimuli (23, 24). They are therefore, an effective tool for developing individual, group or collective tactical aspects in initiation (25) and high-performance training (26).

Although there is an increasing number of studies involving manipulations of SSGs in team sports, there is a paucity of studies that analyzing the external and internal load demands in futsal. To the best of our knowledge, no studies have assessed the external and internal load demands of futsal SSGs. By comprehensively monitoring external and internal load, wellness status and readiness for training and matches, coaches and practitioners can tailor training programs and recovery strategies to optimize team performance (27). Therefore, a better understanding of the relationship between the manipulation of practice tasks, such as the area and the relative area of play per player, in load demands of futsal players is required (4, 15, 28). Understanding the relationship between the demands of each task and competition is crucial to design and management of training programs, particularly at the microcycle level (20). The aim of this study was to analyze how manipulating the space and number of players affects the external and internal load demands of young futsal athletes. It was hypothesized that changing both the space and number of players would result in variations in the external and internal load demands of each task compared to the match. It is suggested that practice tasks with large spaces and a low number of players increase both external and internal load compared to the match. Conversely, tasks with small space per player in relation to the match produce low internal and external load in futsal players.

## Methods

### Sample

The sample size was calculated using G*Power version 3.1.9.7 (Heinrich-Heine Universität Düsseldorf—Düsseldorf, Germany), using the paired *t*-test with the following input parameters: (i) one-tailed (based on pilot study data); (ii) large effect size (≥0.50) as described by Cohen (29) [also based on pilot study data (0.58)]; (iii) α = 0.05; and (iv) β = 0.95, both (iii and iv) according to Field (30), indicating a minimum sample size of 20 individuals.

To meet this assumption, a sample of 42 male youth players (U17), from three futsal teams competing in the Castelo Branco (Portugal) district championship have been selected (age = 15.62 ± 0.58 years; height = 173 ± 5.90 cm; weight = 63.55 ± 10.24 Kg; %body fat = 9.30 ± 4.37%; futsal experience = 4.79 ± 3.21 years). All the players from the team were included in the study. The exclusion criteria were as follows: (i) those who, due to injury, did not participate in all phases of the research; and (ii) voluntarily, withdrew from participating in the study.

In terms of tactical positions, 14.29% were *goalkeeper (GK)* (*n* = 6), 14.29% were *defender* (*n* = 6), 47.62% were *wingers* (*n* = 20), 19.05% were *pivots* (*n* = 8) and 4.76% were *universal* (*n* = 2). In terms of lower limb dominance, most of the players were right-handed (88.10%). Informed and written consent was provided by the club, the head coach, the players, and their legal guardians before the start of the data collection. The study protocol adhered to the guidelines of the ethics committee of the local university and the recommendations of the Declaration of Helsinki. The study protocol was approved by the Ethics Committee of University of Beira Interior (CE-UBIPj-2018–029).

### Experimental design

**Figure 1:**
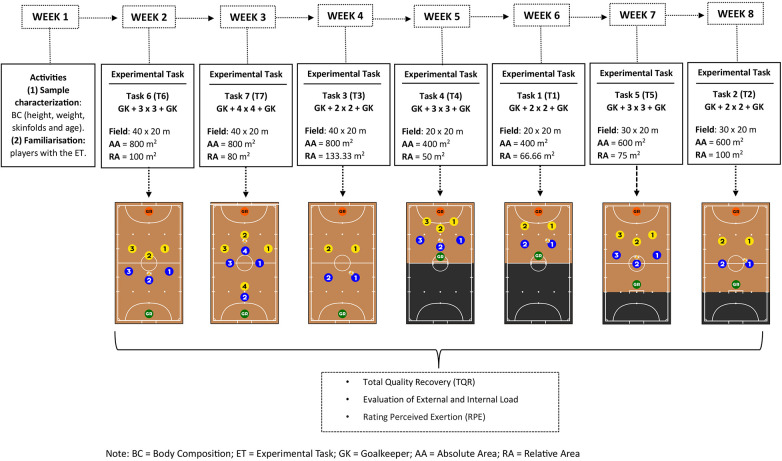
Representation of experimental design.

This cross-sectional study lasted 8 weeks and was carried out between February and April 2023 (Figure 1). In the first week, individuals were familiarized with the intervention procedures and evaluations were carried out to characterize the player's sample. In the following eight weeks, interventions were carried out through the practice of six futsal tasks (T1, T2, T3, T4, T5, T6) and a futsal match (T7), played under the official rules of the sport (31). The futsal tasks (T1–T6) were manipulated considering the: (i) number of players involved in the task; (ii) the dimensions of the playing field in terms of absolute area (AA) (length x width, respectively) and relative area (RA = AA ÷ number of players involved in the task). All the tasks were carried out in the presence of GKs and regular goals. Before the tasks, coaches instructed the teams to adopt a pressure individual defence over all the field.

The tasks were carried out on the pitch where the teams trained regularly. The order of the tasks and the composition of the players per team were randomly selected using the digital tool random.org (School of Computer and Statistics, Trinity College—Dublin, Ireland). Consequently, the timeline unfolded as follow: The futsal task T6 was experienced in week 2, T7 in week 3, T3 in week 4, T4 in week 5, T1 in week 6, T5 in week 7 and T2 in week 8 (Figure 1). Each team, participated in a specific training session with a total duration of 40 min, structured temporally as follows: (i) a standardized warm-up lasting 10 min, with the execution of tactical-technical actions with the ball: passing, receiving, ball control, dribbling and shooting; (ii) practice of the tasks over 20 min, divided between action and recovery. The total action time was 8 min, divided into 4 repetitions lasting 2 min. To reduce the time of stoppages, when one ball was out, or a goal was scored, the goalkeeper of the team in possession introduced a new ball in play. The effective time of play correspond to approximately to 8 min in every task. The total recovery time between repetitions was 12 min, with a 3 min recovery interval between each one; (iii) stretching exercises lasting 10 min. All the players in the sample experienced each of the seven tasks in the experimental design for the same amount of time. During the research, the three teams were playing the same regional level competition in the 2022–2023 season. All the teams practiced twice a week, with a 48 h break between training sessions, and played an official match on Saturdays. Each team experienced one experimental task per week during the training sessions. The data was collected in the evening, between 7 pm and 8.30 pm, in the following order: Team 1 (Mondays), Team 2 (Tuesdays) and Team 3 (Wednesdays). Data was collected with a mean temperature of 15° (Min: 10° to Max: 22°) and mean humidity of 61% (Min: 37% to Max: 87%).

Before starting each training session, it was verified the state of readiness and recovery of participants through total quality recovery Scale (TQR) using a 0- to 10-point scale (32). Players with a TQR higher than score 5 (“adequate recovery”), were considered fit, and were able to participate in the training session. All the players performed an adequate recovery. The TQR were as follows: T1 (7.45 ± 0.86); T2 (7.02 ± 0.92); T3 (7.17 ± 1.01); T4 (7.10 ± 0.85); T5 (6.95 ± 1.03); T6 (6.83 ± 1.03); and T7 (6.74 ± 0.99). The data was collected by the first author that have more than 10 years of experience in monitoring procedures in futsal teams.

#### Physical performance—external and internal load

**Figure 2:**
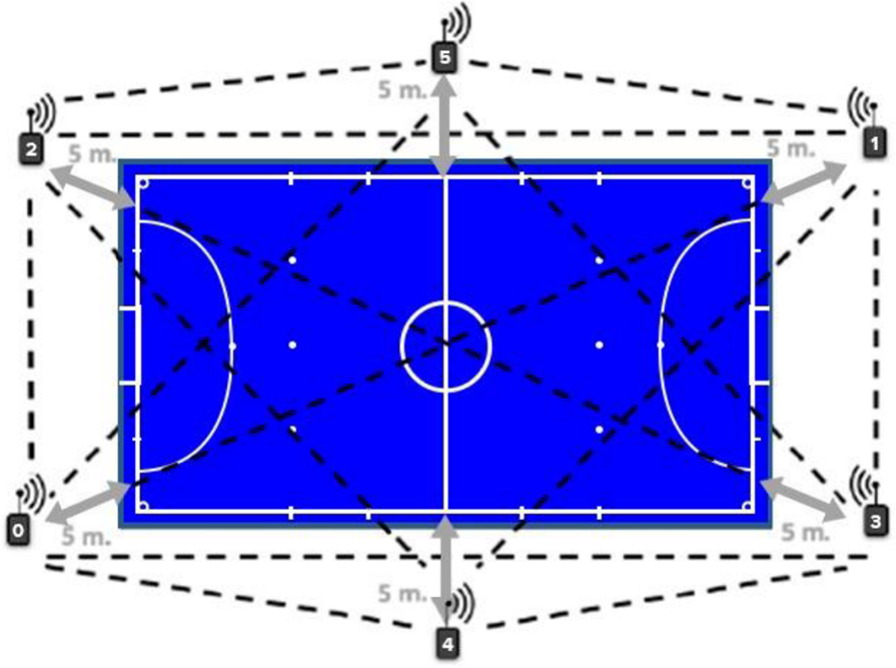
Distribution of LPS antennas and reference distance for the futsal court. Source: Adapted from Serrano et al. (7).

To measure external and internal load during futsal tasks, the WIMU PRO™ Ultra-Wideband (UWB) tracking system (Realtrack Systems, Almeria, Spain) was used. This Local Positioning System (LPS) consists of six UWB antennas, which were arranged outside the playing court, and operates using triangulation between the antennas and the units to derive the *X* and *Y* coordinates of each unit (Serrano et al., 2020) (Figure 2). The devices were turned on 10 min before the warm-up and placed on the players who wore a customized and specific neoprene vest, located in the midline between the scapulae, at the level of the seventh cervical vertebra (C7). The data, from the beginning to the end of each task, excluding the recovery interval, were analysed using SPRO software (Realtrack Systems SL, Almeria, Spain). The sampling frequency was 18 Hz, with 18 records per second. The accuracy and reliability of these devices have been previously reported and validated (33).

From the positional data, variables were extracted based on two main external load categories: kinematics and mechanical (34). The absolute values of each variable were calculated (3, 7).

Regarding internal load (Table 1), a GARMIN HR (Garmin Ltd., Olathe, Kansas, United States) monitoring band was used by the players (35). HR was measured in relation to each task. For that peak, mean and percentage of peak values in the task were registered (9). The RPE and ssession rating perceived exertion (s-RPE) was measured (36). RPE was quantified using the CR-10 scale (12). This scale was applied individually thirty minutes after the end of the training session, and the participants answered the question: “How was your training session?”, rating the session in a quantitative score between 0 and 10, where the maximum value (10) corresponds to the greatest physical effort, and the minimum value (0) corresponds to absolute rest. This scale was always applied by the same researcher, who repeated the assessment protocol described after each of the seven tasks had been completed. The s-RPE was measured by multiplying the RPE score by the total duration of the session and quantified in Arbitrary Units—AU (36).

**Table 1:** External load and internal load variables measured.

Category	Variable	Units	Description
External Load			
Kinematics	Total distance (TD)		Total distance covered (m)
	Relative distance (RD)	meters (m)	[18.1, 21 Km/h]
			[21.1, 30 Km/h]
Mechanical	Total distance at High Intensity Accelerations (HIAC)	meters (m)	>3 m/s^2^
	Total distance at High Intensity Decelerations (HIDC)		<−3 m/s^2^
Internal Load			
Heart rate (HR)	HR peak (HR_Peak_)	beats per minute (bpm)	Maximum value of HR in the taks
	HR average (HR_Avg_)		Mean value of HR in the task
	% HR maximum (%HR_Max_)	meters (m)	% of maximum value reached in the task
	Relative % of HR		[>85%] (m)
Rating of Perceived Exertion (RPE)	RPE	A.U.	RPE
	s-RPE	A.U.	RPE × Time of session

A.U., Arbitrary Units.

**Table 2:** Comparison of the effect of manipulating the playing space and the number of players on external load variables (*n* = 36).

		GK + 2 × 2 + GK			GK + 3 × 3 + GK			GK + 4 × 4 + GK
		20 × 20	30 × 20	40 × 20	20 × 20	30 × 20	40 × 20	40 × 20
		T1	T2	T3	T4	T5	T6	T7
		Mean ± SD	Mean ± SD	Mean ± SD	Mean ± SD	Mean ± SD	Mean ± SD	Mean ± SD
		(Mín-Máx)	(Mín-Máx)	(Mín-Máx)	(Mín-Máx)	(Mín-Máx)	(Mín-Máx)	(Mín-Máx)
Total Distance	(m)^a^	862.98 ± 75.64^¶^	983.63 ± 94.97*^,^^†^	1,098.50 ± 103.03*^,^**^,^^†^^,^^‡^^,^^¶^	826.87 ± 88.01^¶^	968.03 ± 63.22*^,^^†^^,^^¶^	1,033.47 ± 88.11*^,^**^,^^†^^,^^‡^^,^^¶^	918.94 ± 61.56
		(729.60–1,007.20)	(762.70–1,193.60)	(879.80–1,293.50)	(669.40–982.30)	(854.40–1,110.80)	(833.50–1,228.60)	(822.50–1,036.70)
Relative Distance (m)								
[18,1–21,0]	km/h^b^	14.16 ± 10.14**^,^***^,^^‡^^,^^§^^,^^¶^	51.80 ± 24.22	87.50 ± 28.50^¶^	11.64 ± 10.73**^,^***^,^^‡^^,^^§^^,^^¶^	32.68 ± 16.83^,^***	65.50 ± 22.28	35.27 ± 15.93
		(0.00–43.60)	(9.60–106.00)	(37.00–141.50)	(0.00–56.40)	(1.70–84.00)	(26.10–141.20)	(2.40–86.60)
[≥ 21,1]	km/h^b^	2.27 ± 3.50^¶^	19.38 ± 15.16*^,^^†^	44.32 ± 35.45*^,^^†^^,^^‡^^,^^¶^	11.54 ± 2.93^¶^	5.21 ± 5.55	36.11 ± 23.23*^,^^†^^,^^‡^	12.95 ± 14.39
		(0.00–14.10)	(0.30–59.00)	(3.70–147.50)	(0.00–15.40)	(0.00–21.80)	(1.40–87.30)	(0.00–80.50)
*HIAC*	(m)^a^	215.65 ± 30.98	280.10 ± 37.74*^,^^¶^	272.14 ± 35.38>*^,^^†^^,^^‡^^,^^¶^	220.23 ± 57.40	240.79 ± 35.20*^,^^¶^	253.46 ± 39.95*^,^^¶^	219.73 ± 30.76
		(144.19–283.70)	(197.35–352.32)	(200.74–338.57)	(123.19–333.20)	(182.47–316.98)	(144.71–330.46)	(162.06–285.64)
*HIDC*	(m)^a^	206.62 ± 35.66	287.18 ± 44.54*^,^^†^^,^^§^^,^^¶^	256.64 ± 37.85*^,^^¶^	215.85 ± 58.88	237.53 ± 37.08*^,^^¶^	236.40 ± 37.32*^,^^¶^	211.71 ± 28.48
		(132.25–279.77)	(191.88–364.94)	(188.21–322.20)	(108.18–324.02)	(168.29–303.28)	(151.50–306.43)	(170.38–281.61)

SD, standard deviation; TD, Total distance; HIAC, High intensity accelerations; HIDC, High intensity decelerations.

^a^ ANOVA.

^b^ Friedman Test.

* (*p* < 0.05) in relation to T1.

** (*p* < 0.05) in relation to T2.

*** (*p* < 0.05) in relation to T3.

^†^ (*p* < 0.05) in relation to T4.

^‡^ (*p* < 0.05) in relation to T5).

^§^ (*p* < 0.05) in relation to T6.

^¶^ (*p* < 0.05) in relation to T7.

### Statistical analysis

To analyse the external and internal load variables, only the field players were included (*n* = 36). Descriptive analysis was used to calculate minimum and maximum values, mean and standard deviation of all the variables considered. Analysis of variance (ANOVA) for repeated measures with Bonferroni post-hoc for variables with normal distribution and Friedman test for those without normal distribution with Bonferroni post-hoc were used to compare the tasks. Significance was set at *p* ≤ 0.05. All analyses were performed using the Statistical Package for Social Sciences (IBM SPSS, IBM Corporation, Armonk, NY, USA, 25.0).

## Results

The absolute results of external load revealed an interaction between the task constraints for total distance [*F* (6.00) = 63.033; *p* < 0.001]. In relation to T7 (the match format), T3 (*p* = 0.001), T5 (*p* = 0.035) and T6 (*p* = 0.001) showed higher total distance covered values, while T1 and T4 (*p* = 0.001) showed lower values. Similarly, T3 and T6 showed higher total distance covered values compared to T1, T2, T4, T5 (*p* = 0.001), and T2 and T5 compared to T1 and T4. In general, T3 and T6 showed the highest values, while T1 and T4 showed the lowest (Table 2).

A significant interaction emerged for relative distance covered at speeds ranging from 18.1–21.0 Km/h among the various tasks [*X*² (6) 116.051; *p* < 0.001]. In relation to T7 (the match format), only T3 (*p* = 0.001) showed higher distance values, while T1 and T4 (*p* = 0.018; *p* = 0.006) showed lower values. Similarly, there were higher distance covered values in T3 compared to T1, T4 and T5 (*p* = 0.001) and in T2, T5, T6 compared to T1 and T4 (*p* = 0.05). In general, T3 showed the highest distance values between 18.1 and 21.0 km/h while T1 and T4 showed the lowest values (Table 2).

For relative distance covered at speeds greater than 21.1 Km/h, there was an interaction between the tasks [*X*² (6) 107.077; *p* < 0.001]. In relation to T7 (the match format), only T3 (*p* < 0.05) showed distance covered values higher than 21.1 km/h, while T1 and T4 (*p* < 0.05) showed lower values. When comparing tasks, there were higher distance covered values in T3 compared to T1, T4 and T5 (*p* = 0.001), in T2 and T6 compared to T1 and T4 (*p* = 0.001), and in T6 compared to T5 (*p* = 0.001). In general, T3 showed higher values, while T1 and T4 showed lower values (Table 2).

The analysis of HIAC, revealed an interaction between tasks [*F* (2.909) = 12.128; *p* < 0.001]. In relation to T7 (the match format), T2, T3 (*p* = 0.001) and T5 and T6 (*p* < 0.05) showed higher HIAC values. Similarly, higher HIAC values were observed in T3 compared to T1 (*p* = 0.001), T4 (*p* = 0.038) and T5 (*p* = 0.001), and in T2, T5 and T6 compared to T1 (*p* < 0.05). In general, T2 and T3 showed the highest values while T1 and the match showed the lowest values (Table 2).

For HIDC, results revealed an interaction between tasks [*F* (2.619) = 12.304; *p* < 0.001]. In relation to the T7 (the match format), T2, T3 (*p* = 0.001) and T5 and T6 (*p* < 0.05) showed higher HIDC values. When comparing tasks, there were higher HIDC values in T2, T3, T5 compared to T1 (*p* = 0.05), in T2 compared to T4 and T6 (*p* = 0.003*; p* = 0.001), and in T6 compared to T1 (*p *= 0.018). In general, T2 and T3 showed the highest values while T1 and the match showed the lowest values (Table 2).

For the internal load variables, there was no interaction between tasks [*F* (4.093) = 3.296; *p* = 0.081] for HR_Peak_. In opposition, there was an interaction between tasks for HR_Avg_ [*F* (3.925) = 6.123; *p* < 0.001] and %HR_Max_ [*F* (3.954) = 6.042; *p* < 0.001]. In relation to T7 (the match format), T1 and T6 (*p* = 0.005; *p* = 0.006) showed higher HR_Avg_ values, as well as higher %HR_Max_. There were no differences between tasks (Table 3). For distance covered above 85% of HR_Max_, there was an interaction between the tasks [*X*² (6) 91.335; *p* < 0.001]. In relation to T7 (the match format), T2, T3, T5 and T6 (*p* = 0.001) showed higher values for distance covered above 85% of HR_Max_. Also, T2, T3 and T6 showed higher values compared to T1 and T4 (*p* = 0.001), and T5 compared to T4 (*p* = 0.004) for distance covered above 85% of HR_Max_.

**Table 3:** Comparison of the effect of space manipulation and number of players on internal load variables (*n* = 36).

		GK + 2 × 2 + GK			GK + 3 × 3 + GK			GK + 4 × 4 + GK
		20 × 20	30 × 20	40 × 20	20 × 20	30 × 20	40 × 20	40 × 20
		T1	T2	T3	T4	T5	T6	T7
		Mean ± SD	Mean ± SD	Mean ± SD	Mean ± SD	Mean ± SD	Mean ± SD	Mean ± SD
		(Mín-Máx)	(Mín-Máx)	(Mín-Máx)	(Mín-Máx)	(Mín-Máx)	(Mín-Máx)	(Mín-Máx)
HR_Peak_ (bpm)^a^		195.53 ± 5.95	193.16 ± 6.01	194.56 ± 5.66	189.97 ± 9.52	194.17 ± 7.22	195.72 ± 6.85	191.35 ± 7.78
		(184.00–209.00)	(181.00–207.00)	(181.00–206.00)	(170.00–208.00)	(179.00–207.00)	(182.00–210.00)	(177.00–208.00)
HR_Avg_ (bpm)^a^		180.63 ± 6.55^¶^	177.16 ± 6.90	176.78 ± 7.25	171.36 ± 12.86	176.23 ± 8.68	177.94 ± 9.14^¶^	169.32 ± 11.06
		(167.00–195.00)	(165.00–191.00)	(157.00–194.00)	(148.00–197.00)	(159.00–193.00)	(159.00–199.00)	(147.00–195.00)
HR_Máx_ (%)^a^		88.37 ± 3.19^¶^	87.03 ± 3.60	86.49 ± 3.58	83.84 ± 6.33	86.22 ± 4.28	87.06 ± 4.45^¶^	82.85 ± 5.43
		(81.86–95.12)	(80.49–93.63)	(76.59–94.63)	(72.20–96.10)	(77.56–94.15)	(77.56–97.07)	(71.71–95.59)
Dist—HR [>85%]	(m)^b^	723.30 ± 84.68	824.68 ± 92.35*^,^^†^^,^^¶^	868.36 ± 185.41*^,^^†^^,^^¶^	629.90 ± 149.92	779.43 ± 96.47^†^^,^^¶^	820.84 ± 138.77*^,^^†^^,^^¶^	556.57 ± 227.59
		(612.77–930.84)	(569.58–1,035.45)	(436.22–1,164.41)	(201.89–870.12)	(513.84–1,012.83)	(584.17–1,148.13)	(119.04–866.32)
RPE (a.u)^b^		4.78 ± 1.34	6.19 ± 1.28*^,^^†^^,^^¶^	8.66 ± 0.65*^,^**^,^^†^^,^^‡^^,^^¶^	4.79 ± 1.24	5.17 ± 1.23	7.19 ± 1.09*^,^^†^^,^^‡^^,^^¶^	4.03 ± 0.87
		(3.00–8.00)	(4.00–9.00)	(7.00–10.00)	(3.00–7.00)	(4.00–8.00)	(5.00–9.00)	(3.00–6.00)
s-RPE (a.u)^b^		191.25 ± 53.51	247.50 ± 51.24*^,^^†^^,^^‡^^,^^¶^	346.25 ± 26.12*^,^**^,^^†^^,^^‡^^,^^¶^	191.52 ± 49.76	206.67 ± 49.36	287.78 ± 43.63*^,^^†^^,^^‡^^,^^¶^	161.18 ± 34.80
		(120.00–320.00)	(160.00–360.00)	(280.00–400.00)	(120.00–280.00)	(160.00–320.00)	(200.00–360.00)	(120.00–240.00)

Data is presented in mean and standard deviation (SD), minimum (Min) and maximum (Max) values.

HR_Peak_, Heart rate peak; HR_Avg_, Heart rate average; RPE, rating of perceived exertion; s-RPE, session rating perceived exertion; Dist, distance.

^a^ ANOVA.

^b^ Friedman Test.

* (*p* < 0.05) in relation to T1.

** (*p* < 0.05) in relation to T2.

*** (*p *<  0.05) in relation to T3.

^†^ (*p* < 0.05) in relation to T4.

^‡^ (*p* < 0.05) in relation to T5.

^§^ (*p* < 0.05) in relation to T6.

^¶^ (*p* < 0.05) in relation to T7.

Regarding RPE there was an interaction between the tasks [*X*² (6) 119.703; *p *< 0.001]. In relation to T7 (the match format), T2, T3 and T6 (*p *= 0.001) showed higher values. When comparing tasks, there were higher RPE for T2 compared to T1 and T4 (*p* = 0.010; *p* = 0.039), for T3 compared to T1, T2, T4 and T5 (*p* = 0.001), and for T6 compared to T1, T4 and T5 (*p* = 0.001). In general, T2, T3 and T6 showed higher values while T7 (the match format) showed the lowest RPE values.

Regarding s-PRE, there was an interaction between the tasks [*X*² (6) 114.536; *p* < 0.001]. In relation to T7 (the match format), T2, T3 and T6 (*p* = 0.001) showed higher values. When comparing tasks, T2, T3 and T6 showed higher values compared to T1 (*p* = 0.001), as well as higher values for T2, T3 and T6 compared to T4 (*p* = 0.039; *p* = 0.001; *p* = 0.001), respectively. T3 and T6 were higher than T5 (*p* = 0.005; *p* = 0.001), respectively and, T3 was superior to T2 (*p* = 0.001). In general, T2, T3 and T6 showed higher values while T1, T4 and T7 showed the lowest s-PRE values.

## Discussion

The aim of this study was to analyze the effects of manipulating space and number of players on the external and internal load demands of youth futsal players. In line with our expectations, variations in the space and number of players involved in the practice tasks promoted general variations in the external and internal load of futsal players. High internal and external loads were observed in the tasks with large spaces and low numbers of players. In terms of the match, the results partially confirmed our hypothesis. Tasks with a small space per player produced particularly low external load, while the variation in internal load was not so clear.

In general, the results show that practice tasks with larger relative areas increase the total distance covered by futsal players. The design of practice tasks, to promote high running volume, high-intensity distances and sprinting should consider larger relative areas with medium to large areas (30 × 20–40 × 20 meters) and GK + 2 vs. 2 + GK or GK + 3 vs. 3 + GK structures. The results clearly show that in order to stimulate speeds above 18 km/h, coaches should use the T3 task, GK + 2 vs. 2 + GK in a 40 × 20 m area. It has one of the largest relative areas (133 m^2^) among the structures studied and produced the most significant player results. The results are clearly in line with the findings of various football studies where similar trends have been observed (37, 38). The studies concluded that practice tasks in futsal played on large pitches were more physically demanding than those played on small pitches. Players covered significantly more distance in all movement categories, including acceleration and deceleration efforts.

Interestingly, and contrary to some expectations, the high intensity accelerations, and decelerations also showed high values in the practice tasks with high areas per player. This means that higher relative areas per player also promoted high mechanical efforts in futsal players. The results of this study showed that the use of fewer players (2 vs. 2 or 3 vs. 3) in high areas, tended to reveal higher distances covered in high intensity accelerations and decelerations. In line with this, as previously proposed, the use of medium to high areas of play with few players can be used to promote high intensity and tactical group actions (20). These findings are in line with previous studies, where researchers have consistently found that reducing the number of players and increasing the field area leads to increased physiological demands (39–41). Thus, the use of a practice task with few players in medium space (GK + 2 vs. 2 + GK in a 30 × 20 m) proved to be the most effective in promoting high intensity accelerations and decelerations. This contradicts the misconception held in some futsal coaching books which suggest that coaches should reduce the size of the pitch and use, for example, half-court (20 × 20 m) to increase the mechanical load and, consequently, the number of accelerations and decelerations. This empirical idea could be justified by the higher number of technical actions required on small pitches (38), which could lead to more neuromuscular fatigue (37, 42, 43). On the other hand, players covered less distance and performed fewer sprints on smaller pitches, which is consistent with the findings of Castillo et al. (44). In their study, a decrease in maximal oxygen uptake was also observed in smaller pitch sizes.

The current question is whether the observed trend in external load also applies to internal load. In other words, do the factors that contribute to the increase in external load have a similar effect on the increase in internal load? Notably, Barbero-Alvarez et al. (9) reported that 83% of futsal match time was spent with at an HR above 85% of HR_max_. Interestingly, our esults suggest that the distance covered above 85% of HR_max_ appears to be the most effective in discriminating intensity among the different practice tasks analysed in this study. This trend is consistent with the relationship observed for external load, where greater relative area correlates with greater internal load. In this context, practice tasks T3 (GK + 2 vs. 2 + GK in 40 × 20 m) and T6 (GK + 3 vs. 3 + GK in 40 × 20 m) proved to be the most demanding in terms of distance covered above 85% of HR_max_. In addition, the highest RPE was recorded in the same practice tasks, suggesting that larger field sizes and greater area per player lead to a higher perception of physical effort among players. In this regard, Aguiar et al. (45) observed that formats with 2- and 3-a-side formats resulted in a higher percentage of HR_max_ values compared to 4- and 5-a-side formats in professional football players, which is consistent with our findings. Additionally, in their study, the authors found that the 2-a-side format had the highest RPE values. Another interesting aspect is that all the different practice tasks analysed show higher internal load values than the T7 (match situation), which is in contrast to the analysis of external load variables. Further research is needed to understand the relationship between such manipulations, the physical and physiological demands and the players' level of practice.

Compared with the match condition, practice tasks with larger relative areas of play tend to have higher external and internal loads, particularly for high intensity actions (distance covered at high intensity actions, sprint and distance covered with HR above 85% of HR_max_). This means that to increase the efforts and the physiological impact of training sessions, coaches should use tasks with few numbers of players in medium to large areas (39–41). Interestingly, in comparison with previous research characterising the demands of futsal in a match context (3), lower values of total distance covered were observed in all the practice tasks performed in this study. However, when comparing the distances covered above 18 km/h, T2/T3 and T6, the tasks with higher relative area than the match showed similar and higher values than those observed in the competitive context of elite players. In opposition, all the practice tasks replicated the physiological demands observed in the competitive context by elite players (the achievement of similar values of HR_max_ > 170 bpm, and %HR_max_ > 80% (8). This means that practising these tasks for two minutes contributed to replicate the physiological demands of the competitive environment. However, to replicate the kinematic of the mechanical demands of the competition (3), particularly the tasks with a large relative area should be used in particular.

Despite the practical implications of this study, some precautions should be taken when generalizing the results. The results sample the practice of U17 teams (regional level) and should be considered in this context. Players with different levels of practice and different ages adapt differently to the same tasks. Therefore, a better understanding of the manipulation of the space and the number of players requires the development of further studies involving a large number of teams involved and, in particular, the comparison between players of different ages and levels of practice. Such knowledge will help to optimize the relationship between player characteristics, goal scoring and player development in futsal.

## Conclusions

In conclusion, rather than looking at size of the pitch or the number of players involved in each practice task, coaches should consider the relative pitch area per player to better understand the physical and physiological demands of futsal. The use of high relative areas per player, through the use of large relative areas (30 × 20 and 40 × 20 m) and a low number of players (2- and 3-a-side), tends to increase the external and internal load on futsal players compared to match demands. Therefore, the definition of the playing areas and the number of players involved should link the physical and physiological effects of the exercises to the tactical objectives. The use of simulated matches or tasks with a high number of players in medium to small spaces tends to promote collective adaptations with low physical and physiological effects. The use of tasks with a low number of players and large areas tends to promote group adaptations with high physical and physiological effects.

## Data Availability

The raw data supporting the conclusions of this article will be made available by the authors, without undue reservation.
